# The complete chloroplast genome of *Cycas bifida,* an extremely small population protected species

**DOI:** 10.1080/23802359.2021.1973919

**Published:** 2021-09-15

**Authors:** Deng Zhang, Yanqiang Cao, Zhaocen Lu

**Affiliations:** Guangxi Key Laboratory of Plant Conservation and Restoration Ecology in Karst Terrain, Guangxi Institute of Botany, Guangxi Zhuang Autonomous Region and Chinese Academy of Sciences, Guilin, China

**Keywords:** Cycas bifida, chloroplast genome Illumina sequencing endangered species

## Abstract

*Cycas bifida* (Dyer) K.D.Hill (2004) is an extremely small population-protected species of China. In this study, we reported the first chloroplast genome sequence of *C. bifida*. The chloroplast genome of *C. bifida* included two single-copy regions (large single-copy (LSC) and small single-copy (SSC)) and a pair of inverted repeats (IRs) regions comprising 88,946 bp, 23,107 bp, and 25,053 bp, respectively. The complete chloroplast genome of *C. bifida* contains 131 genes, including 86 protein-coding genes, 37 transfer RNA genes, and 8 ribosomal RNA genes. The overall GC content of the *C. bifida* chloroplast genome is 39.41%, and the LSC, SSC, and IR regions occupy 38.70%, 36.52%, and 42.02%, respectively. A phylogenetic analysis was performed based on complete chloroplast genomes from 15 species and found that *C. bifida* was closely related to *Cycas szechuanensis* W.C.Cheng & L.K.Fu.

*Cycas bifida* (Dyer) K.D.Hill (2004), an extremely small population-protected species of China, belongs to the cycad family (Cycadaceae, *Cycas*) (Zheng et al. 2018; Lin et al. 2019). Cycads are regarded as the ‘Living Fossils’ and belong to a specialized group of plants having ancient lineage possessing great significance from the evolutionary point of view (Goel and Khuraijam 2015). *Cycas bifida* was listed as rare and endangered plant (Ying 2013), with a geographical range restricted to a total of two known countries (Vietnam and China) (Osborne 1995). However, the complete chloroplast genome of *C. bifida* has not been sequenced.

In this study, we assembled and characterized the complete chloroplast (cp) genome of *C. bifida* and determined its molecular phylogeny and genetic information. Fresh leaf tissues of *C. bifida* were collected from Guangxi Institute of Botany, Guilin, China (GPS: 25°4’44” N, 110°18’1” E). Its voucher specimen was deposited in the herbarium at the Guangxi Institute of Botany (http://www.gxib.cn/spIBK/, Z. C. Lu, email: zhaocenlu@163.com) with the voucher number as IBK00435048. Total genomic DNA was extracted by the modified CTAB method (Doyle 1991). A total of 6 G raw data from Illumina Hiseq Platform were screened and assembled into a complete chloroplast genome by GetOrganelle in a typical way (Jin et al. 2020). The assembled sequences were annotated using the tool CPGAVAS2 and using *Cycas szechuanensis* (NC_042668.1) as reference (Shi et al. 2019, Wang et al. 2018). The annotated chloroplast genome sequence was submitted to the NCBI GenBank database under the accession number MW900434.

Based on the assembling results, the length of the complete chloroplast genome of *C. bifida* is 162,159 bp. The chloroplast genome of *C. bifida* included two single-copy regions (large single-copy (LSC) and small single-copy (SSC)) and a pair of inverted repeats (IRs) regions comprising 88,946 bp, 23,107 bp, and 25,053 bp, respectively. The complete chloroplast genome of *C. bifida* contains 131 genes, including 86 protein-coding genes, 37 transfer RNA genes, and 8 ribosomal RNA genes. The overall GC content of *C. bifida* chloroplast genome is 39.41%, and the LSC, SSC, and IR regions occupy 38.70%, 36.52%, and 42.02%, respectively.

To reveal the phylogenetic position of *C. bifida* with other Cycads, the complete chloroplast genomes of 14 published species within Cycadales and one outgroup *Cupressus chengiana* (NC_034788.1) were downloaded from the NCBI. A total of 83 coding sequences (*accD*, *atpA*, *atpB*, *atpE*, *atpF*, *atpH*, *atpI*, *ccsA*, *cemA*, *chlB*, *chlL*, *chlN*, *clpP*, *infA*, *matK*, *ndhA*, *ndhB*, *ndhC*, *ndhD*, *ndhE*, *ndhF*, *ndhG*, *ndhH*, *ndhI*, *ndhJ*, *ndhK*, *petA*, *petB*, *petD*, *petG*, *petL*, *petN*, *psaA*, *psaB*, *psaC*, *psaI*, *psaJ*, *psbA*, *psbB*, *psbC*, *psbD*, *psbE*, *psbF*, *psbH*, *psbI*, *psbJ*, *psbK*, *psbL*, *psbM*, *psbN*, *psbT*, *psbZ*, *rbcL*, *rpl14*, *rpl16*, *rpl2*, *rpl20*, *rpl22*, *rpl23*, *rpl32*, *rpl33*, *rpl36*, *rpoA*, *rpoB*, *rpoC1*, *rpoC2*, *rps11*, *rps12*, *rps14*, *rps15*, *rps16*, *rps18*, *rps19*, *rps2*, *rps3*, *rps4*, *rps7*, *rps8*, *ycf1*, *ycf12, ycf2*, *ycf3*, and *ycf4*) were extracted, and aligned by using MUSCLE (Edgar 2004). The maximum likelihood (ML) tree with 100 bootstrap replicates was performed with RaxML v 8.2.12 (Stamatakis 2014). From the ML phylogenetic tree, *C. bifida* was closely related to *Cycas szechuanensis.* The complete chloroplast genome of *C. bifida* will provide useful genetic resources for further studying on genetic diversity of this important species and help us protect this extremely endangered plant (Figure 1).

**Figure 1.**
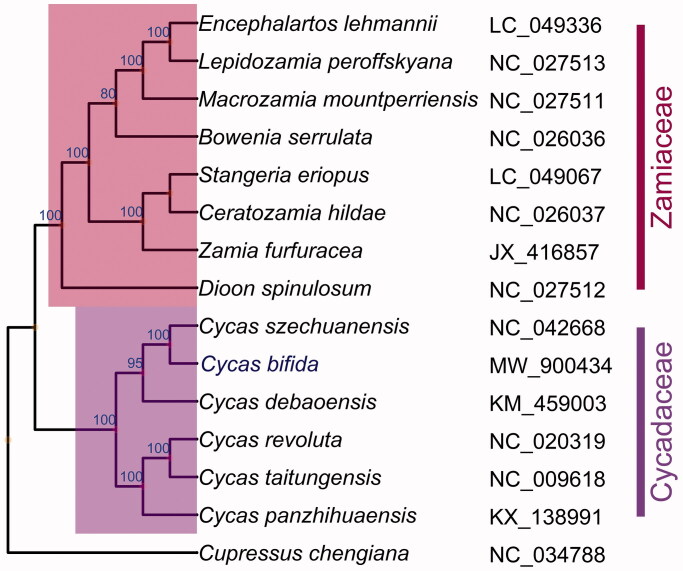
ML phylogenetic tree based on chloroplast gene sequences of *C. bifida* and other 14 species. Numbers in the nodes are the bootstrap values from 100 replicates.

## Data Availability

The genome sequence data that support the findings of this study are openly available in GenBank of NCBI at (https://www.ncbi.nlm.nih.gov/) under the accession no. MW900434. The associated BioProject, SRA, and BioSample numbers are PRJNA723747, SRX10656572, and SAMN18828092 respectively. Treefile of 15 species and genes for phylogenetic analysis were deposited at Figshare: https://doi.org/10.6084/m9.figshare.14784537.v5.
